# Convenient conversion of hazardous nitrobenzene derivatives to aniline analogues by Ag nanoparticles, stabilized on a naturally magnetic pumice/chitosan substrate†

**DOI:** 10.1039/d0ra08376c

**Published:** 2020-12-08

**Authors:** Reza Taheri-Ledari, Seyedeh Shadi Mirmohammadi, Kobra Valadi, Ali Maleki, Ahmed Esmail Shalan

**Affiliations:** Catalysts and Organic Synthesis Research Laboratory, Department of Chemistry, Iran University of Science and Technology (IUST) Tehran 16846-13114 Iran maleki@iust.ac.ir +98-21-73021584 +98-21-77240640-50; Central Metallurgical Research and Development Institute (CMRDI) P.O. Box 87, Helwan Cairo 11421 Egypt a.shalan133@gmail.com; BCMaterials, Basque Center for Materials, Applications and Nanostructures Martina Casiano, UPV/EHU Science Park, Barrio Sarriena s/n Leioa 48940 Spain

## Abstract

Herein, silver nanoparticles (Ag NPs), as an effective catalyst for the reduction process of nitrobenzene derivatives to non-hazardous and useful aniline derivatives, are conveniently synthesized on an inherently magnetic substrate. For this purpose, an efficient combination of volcanic pumice (VP), which is an extremely porous igneous rock, and a chitosan (CTS) polymeric network is prepared and suitably used for the stabilization of the Ag NPs. High magnetic properties of the fabricated Ag@VP/CTS composite, which have been confirmed *via* vibrating-sample magnetometer (VSM) analysis, are the first and foremost advantage of the introduced catalytic system since it gives us the opportunity to easily separate the particles and perform purification processes. Briefly, higher yields were obtained in the reduction reactions of nitrobenzenes (NBs) under very mild conditions in a short reaction time. Also, along with the natural biocompatible ingredients (VP and CTS) in the structure, excellent recyclability has been observed for the fabricated Ag@VP/CTS catalytic system, which convinces us to do scaling-up and suggests the presented system can be used for industrial applications.

## Abstract 2

Silver nanoparticles (Ag NPs), as an effective catalyst for the reduction process of nitrobenzene derivatives to non-hazardous and useful aniline derivatives, are conveniently synthesized on an inherently magnetic substrate.

## Graphical abstract

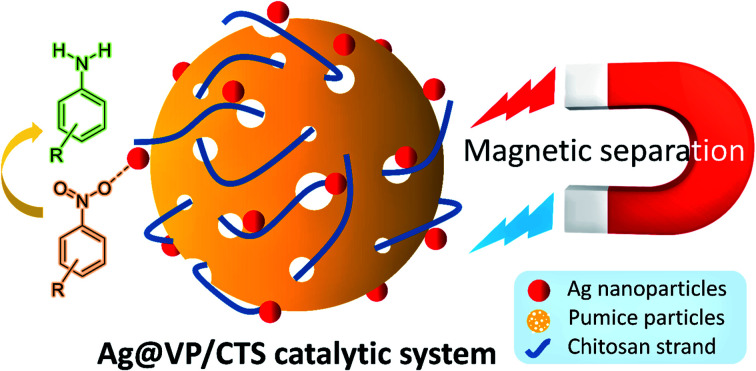

## 1. Introduction

Recently, the design and development of heterogeneous catalytic systems has attracted much attention because they have a great potential advantage of recyclability over the homogenous types.^1^ By immobilization of metallic particles into various combinations of organic and inorganic materials like polymers and clay resources, the scope of the heterogeneous catalytic systems has been amazingly developed.^2–4^ Chitosan (CTS) as a natural polysaccharide with alkaline substances is extracted from chitin shells of crustaceans.^5^ So far, CTS has been widely used for medicinal applications like controlled drug release systems,^6^ skin substitutes,^7^ vaccine delivery,^8^ antibacterial agents,^9^ and wound dressings.^10^ Also, it is a conventional natural metal ion absorbent and is a great candidate for catalytic applications.^5^ Through the addition of some other characteristic properties like magnetism, recyclability of the heterogeneous catalyst will be significantly facilitated.^11^ By only applying an external magnet from the outside part of the flask, the catalyst could be conveniently separated and reused. Previously, so many magnetic catalytic systems in which iron oxide nanoparticles play the crucial role as the magnetic core of the whole structure have been reported by our research group.^12–23^ Also, we have used the mentioned particles as the drug delivery systems with high efficiency,^24,25^ due to its non-toxicity and biodegradability.^26^

Pumice is a volcanic stone that includes numerous voids in its structure, and since it is fallen into the category of the light-weight materials, it can be well dispersed/submerged in the reaction mixture.^27^ The volcanic pumice (VP) consists of internal particles, totally separated from outside, and external particles linked to the surface resembling a sponge.^28^ Generally, porous compounds such as metal–organic frameworks, pumice particles, carbon nanotubes, and graphene oxide are promising materials in the catalytic applications due to their broad surface area, which increases the catalytic performance and efficiency.^29,30^ In the case of the VP, the noticeable inherent magnetic property makes it more distinguished from other types of the porous materials applicable for the catalytic aims.^31^ As discussed above, inherent magnetic property of the VP provides ultra-affluence in the separation as well as the purification processes. Hence, we decided to continue our previous studies on the VP by using that as an appropriate substrate for metal ion stabilization and further catalytic purposes.^32–35^ In this work, we intend to prepare another efficient catalytic system based on a completely natural matrix (including VP and CTS), and apply that for the reduction reaction of the nitrobenzene (NB) derivatives. In addition, the NB derivatives are widely used in the pharmaceutical processes or in producing dyes.^36^ These derivatives are extremely hazardous compounds toward humans and other environmental organisms, and they are barely degraded in the biological processes.^37–39^ So far, so many various strategies have been designed and reported for the reduction pathway of the NB derivatives to converted them to their aniline analogues.^40^ To name a few, the electrochemical approach,^41^ photocatalyst technique,^42^ and the catalytic systems based on the noble or transition metals^43^ are the most common strategies. All of the mentioned strategies included few drawbacks that needed to be modified and improved. For instance, in order to apply catalytic system based on transition metals for reduction reactions, elevated pressure and temperature is required.^44^ Moreover, low current efficiency is the main disadvantage of electrochemical approach.^40^

So far, it has been revealed that silver nanoparticles (Ag NPs) are among the best competent candidates in the catalytic processes due to including impressive electronic and optical properties.^45^ Moreover, the Ag NPs have a considerably high surface to volume ratio that changes their chemical, biological and physical properties.^46^ According to the literature and as our previous experiences disclosed, the Ag NPs include high surface energy that leads them to be quickly aggregated.^21,47^ This property makes the Ag NPs as a significant reducing agent in the catalytic applications. Additionally, the immobilization of the Ag NPs on a polymeric substrate like CTS, enhances their agglomeration.^48–50^

Herein, a novel heterogeneous catalyst is prepared by using of the VP particles and the CTS strands, as a natural-based substrate for the stabilization of the Ag ions, and conversion to the pure metallic NPs. From the architecture aspect, this is demonstrated that the Ag NPs are uniformly synthesized (equal in size and morphology) and finely distributed onto the VP/CTS substrate, *via* an *in situ* process. Then, the magnetic behavior and other critical characteristic properties of the prepared Ag@VP/CTS catalytic system such as average size, porosity, chemical state of the present, metallic elements, and thermal stability are carefully investigated by using different analytical methods. Next, the catalytic performance as well as the reusability behaviors of the Ag@VP/CTS catalytic system are carefully monitored in the reduction reactions of the NB derivatives. In summary, it was revealed that more than 95% of the reaction yield is obtained during only 8 minutes reaction time, at room temperature, through applying the Ag@VP/CTS catalytic system. Besides, this is exhibited that the presented catalytic system is reused for 12 successive runs (by magnetic separation). Ultimately, a feasible mechanism aimed at the catalyzed reduction of the NB derivatives is suggested.

## 2. Result and discussion

### 2.1. Preparation of the Ag@VP/CTS catalyst

**Fig. 1:**
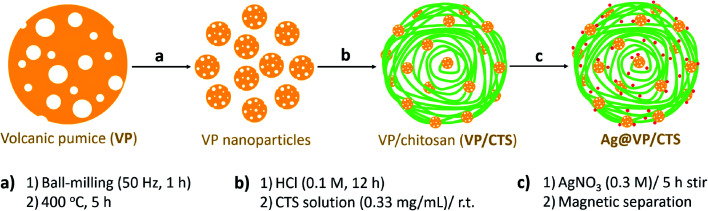
Schematic diagram of the synthetic route of the Ag@VP/CTS nanocomposite.

The preparation route of the Ag@VP/CTS catalyst is given in Fig. 1. Initially, the VP is well grinded and converted to a very fine powder *via* ball-milling technique. For enhancing and depleting the pumice pores from the unwanted fillers, the calcination process was applied using a furnace.^51^ Afterward, the uniformed pumice particles were washed with hydrochloric acid, and dispersed in a solution of the concentrated CTS *via* ultrasonication, under a mild condition. The mixture was washed for removing the excess CTS, and the silver nitrate salt was added to the mixture, which was stirred at room temperature. Finally, the obtained magnetic particles were collected together and washed with distilled water for several times and then dried in the oven for 1 h. The synthesized composite is founded to be exceptionally stable as a result of the presence of numerous –OH groups on both VP and CTS structures, resulting in the powerful H-bindings. Previously, so many methods have been reported in which the Ag ions were stabilized and reduced to the Ag NPs by using of the different polymeric species such as polyvinyl alcohol,^52^ polyethylene glycol,^53^ and polyvinyl chloride.^54^

### 2.2. Characterization

**Fig. 2:**
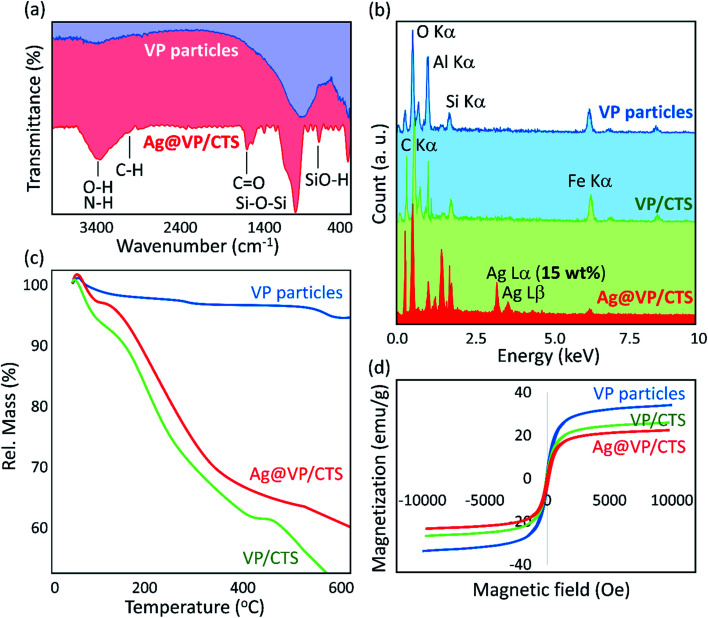
(a) FT-IR spectra, (b) EDX spectra, (c) TGA curves, and (d) VSM curves (at room temperature) of the individual VP nanoparticles (blue line), VP/CTS (green line), and the prepared Ag@VP/CTS nanocomposite (red line).

In order to perform the characterization stage, Fourier-transform infrared (FT-IR) spectra of the neat VP, and Ag@VP/CTS nanocomposite catalyst were provided. As Fig. 2a shows, the peaks related to the stretching vibrations of Si–O–Si and O–H bands at *ca.* 1100 and 3400 cm^−1^, respectively, have been appeared sharper after the calcination process. It can be attributed to the removal of the unwanted fillers in the pores of the VP.^51^ After combination of the CTS, a sharp peak appeared at about 2900 cm^−1^, which origins from the C–H bonds with hybridization state sp^3^. This peak proves successful composition of the CTS, as the organic component in the structure. Also, the peaks appeared at *ca.* 3400 cm^−1^ and 1650 cm^−1^ are belonging to N–H and C=O bonds, respectively, which present in the CTS's structure.^55^ The confirmation of the existence of all the essential elements was done by energy-dispersive X-ray (EDX) spectroscopy (Fig. 2b). Furthermore, in comparison to the individual VP, the synthesized VP/CTS and Ag@VP/CTS composites have shown sharper peak for the carbon element that approving the successful addition of the CTS to the structure. Also, the EDX spectrum of the Ag@VP/CTS nanocatalyst verifies the presence of the Ag element in the final composition structure.

Moreover, the thermal stability of the Ag@VP/CTS nanocatalyst was associated with the neat VP *via* thermogravimetric analysis (TGA), under the air atmosphere in a thermal range of 50 to 600 °C (Fig. 2c). As the TGA curves demonstrate, in all three curves, between *ca.* 60 and 120 °C, a gradual weight loss has emerged that is attributed to the separation of the captured water molecules in the silica network of the VP, which performances as an excessive molecular sieve.^56^ In the range of 120 to 500 °C, an excellent weight loss has been appeared for the curve related to the VP/CTS and Ag@VP/CTS composites, which is accredited to the loss of the –OH groups present in the CTS structure. According to the literature, at this range, the hydroxyl groups of the polymeric chains are removed as the hydrate molecules.^23^ Afterward, in the curve of Ag@VP/CTS, the Ag NPs and then the VP start to decomposition. The inherent magnetic performance of the neat VP particles, VP/CTS, and Ag@VP/CTS composite was also measured *via* vibrating-sample magnetometer (VSM) machine. As is observed in Fig. 2d, the hysteresis curves of the Ag@VP/CTS nanocatalyst demonstrates about *ca.* 10.0 emu g^−1^ decrease in the magnetic saturation, which is quite reasonable since all constitutes of the nanocatalyst don't have magnetic properties. As is observed in the magnetic–hysteresis curves, the magnetic behavior of the VP/CTS composite has not been significantly reduced after addition of the Ag particles. Through this great magnetic property, the Ag@VP/CTS particles could be collected without difficulty through applying of a magnet, and reapplied for several times.

**Fig. 3:**
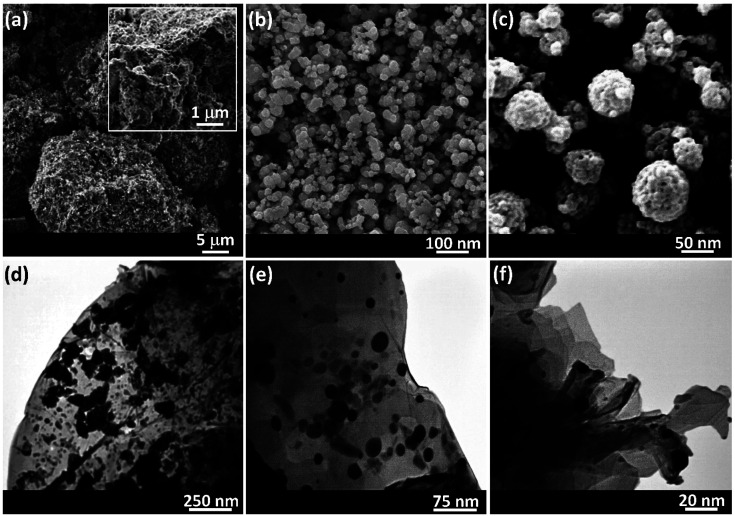
SEM image of: (a) VP powder, (b) VP nanoparticles (prepared *via* ball-milling), and (c) Ag@VP/CTS nanocomposite, and TEM images of the Ag@VP/CTS nanocomposite (d–f).

Microscopic images were checked by scanning electron microscopy (SEM) and transmission electron microscopy (TEM) for exploring the morphology as well as the size of the synthesized Ag@VP/CTS composite (Fig. 3). Image (3a) is appointed to the VP powder and image (3b) is related to the VP NPs after grinding *via* ball-milling. It was evident that the plates are detached and well-dispersed, while they are in agglomerated form before grinding process. Image (3c) is related to the Ag@VP/CTS nanocomposite. As shown, the spherical Ag NPs and the combined CTS strands with VP particles are easily identified. Finally, the TEM image illustrates the formation of the Ag NPs appeared by dark spots in the image. Also, well distribution of these metallic particles on to the VP/CTS surfaces is verified (images 3d–f). Generally, this is clearly observed in the SEM and TEM images that the dominant phase onto the surfaces is formed by the Ag NPs.

**Fig. 4:**
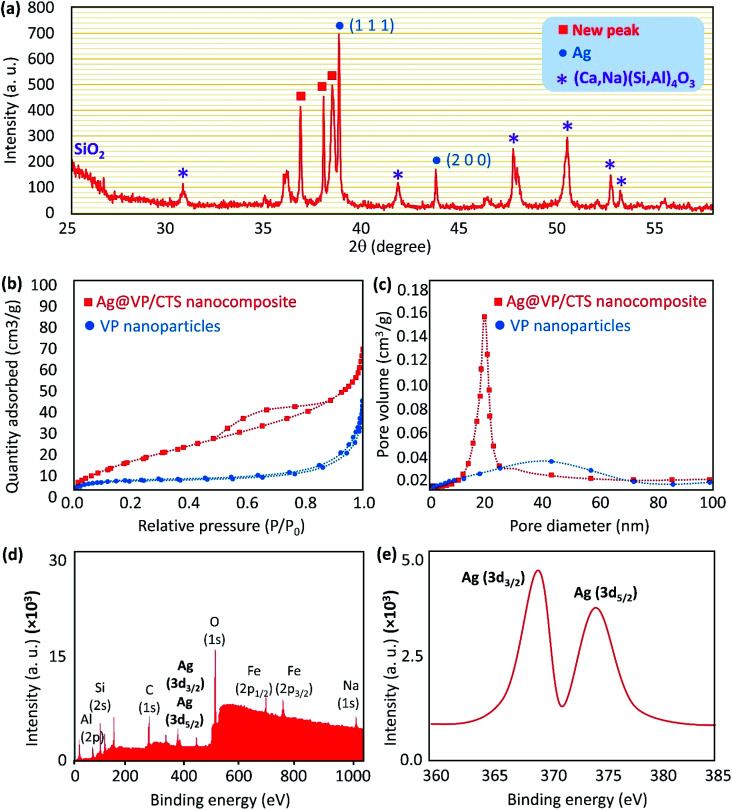
(a) XRD pattern of the Ag@VP/CTS nanocomposite, (b) BET curves, (c) the average pore volume of the individual VP nanoparticles and the fabricated Ag@VP/CTS nanocomposite, *via* adsorption/desorption of N_2_, under ambient conditions, and (d and e) XPS spectra of the Ag@VP/CTS nanocomposite.

Hence, it is quite reasonable to expect that the X-ray diffraction (XRD) pattern of the Ag@VP/CTS nanocomposite be similar to the individual Ag NPs. In this regard, the XRD pattern of the Ag@VP/CTS nanocomposite was prepared and compared with the pattern of the individual Ag NPs, based on the database of JCPDS (PDF#87–0597) (Fig. 4a).^57^ Accordingly, the peaks emerged at 38.07, 44.26 are attributed to the Ag NPs signing by their Miller indices of (1 1 1), and (2 0 0), respectively. The broad peak appeared at *ca.* 25–30° is related to the amorphous structure of the silica network present in the VP's structure.^12^ Also, peaks are observed in the pattern at *ca.* 32°,43°, 51°, 53°, and 54° are assigning to the (Ca,Na)(Si,Al)_4_O_3_.^58^

For confirming the porosity of the synthesized composite *via* adsorption/desorption of N_2_ gas Brunauer–Emmett–Teller (BET) surface area analysis was detected in Fig. 4b and c. The physisorption isotherms of the VP after calcination and Ag@VP/CTS composite is shown. The VP NPs curve Fig. 4b indicates type IV isotherm of mesoporous materials. As shown in the curves, although some internal pores are filled after the addition of CTS and Ag, there are still large internal pores for incorporating the catalytic reaction of the NB reduction. Based on the curves, the pore size range has decreased from 40 nm for pumice powder to 20 nm belonging to Ag@VP/CTS composite. Moreover, the specific surface area also follows the same trend. Results confirmed the occupation of some of the internal pores after composition with CTS and Ag nanoparticles.^59,60^ Also, X-ray photoelectron spectroscopy (XPS) was employed to characterize the valence state of the Ag element (Fig. 4d and e). As expected, two peaks related to Ag (3d_3/2_) and Ag (3d_5/2_) were detected at *ca.* 368 and 374 eV, respectively, indicating Ag^0^ state (metallic nanoparticles).^61^

### 2.3. Application

#### 2.3.1. Optimization of the catalytic system in the reduction of NB derivatives

For reaching the optimized condition, the catalytic performance of the prepared Ag@VP/CTS was explored. For this purpose, different catalytic amounts of the nanocomposite, and amount of the used hydrazine in the reduction reaction of the nitrobenzene were precisely monitored. The detailed information of this experiment is reported in Table 1. As claimed by the table, to reveal the importance of the VP porous structure in the catalyst, the sole Ag/CTS was also applied under the same conditions during the reduction reaction. As can be detected in Table 1 (entry 11), the obtained reaction yield is reduced after removing the VP particles from the catalytic system. Moreover, since Ag is the main catalyst site of this catalytic system it is estimated that by removing Ag from the catalytic system, yield of the reaction would be significantly deducted (see Table 1 entry 2). From the table, it has been revealed that the optimum conditions were provided by using 0.03 g of the Ag@VP/CTS catalyst during 5 min stirring, at room temperature (Table 1, entry 5).

**Table 1:** Reaction optimization by utilizing different amounts of nanocatalyst and N_2_H_4_·H_2_O, in ethanol and reflux conditions in reduction reaction of NBs

Entry	Cat.	Cat. amount (mg)	N_2_H_4_ (mol%)	Time (min)	Yield^a^ (%)
1	—	—	5	15	Trace
2	VP@CTS	30	5	5	20
3	Ag@VP/CTS	10	5	5	85
4	Ag@VP/CTS	20	5	5	91
5	Ag@VP/CTS	30	5	5	95*
6	Ag@VP/CTS	40	5	5	95
7	Ag@VP/CTS	50	5	5	95
8	Ag@VP/CTS	30	4	5	92
9	Ag@VP/CTS	30	2	5	85
10	Ag@VP/CTS	30	1	5	70
11	Ag/CTS	30	5	5	94

a *Optimum condition using the Ag@VP/CTS nanocomposite (30.0 mg), nitrobenzene (1.0 mmol), N_2_H_4_·H_2_O (5.0 mol%), and ethanol (2.0 mL), at 70 °C.

#### 2.3.2. Catalyzed synthesis of the aniline derivatives

The catalytic efficiency of the prepared Ag@VP/CTS composite was also investigated by utilizing the various NB derivatives as founded in Table 2. Surprisingly, in a brief period by using Ag@VP/CTS in the reaction, high yield was obtained. Comparing the results to other synthesized catalysts, the results reveal that the prepared Ag@VP/CTS is a great candidate for NB reduction reactions. Additionally, the NMR data of all of the synthesized aniline derivatives are founded in Fig. S1–S10, in the ESI.†

**Table 2:** Gained yields after the reduction of NB derivatives to aniline analogues under optimized condition

Entry	NB structure	Product	Time (min)	Yield (%)	Mp (°C)	
					Found	Reported
1	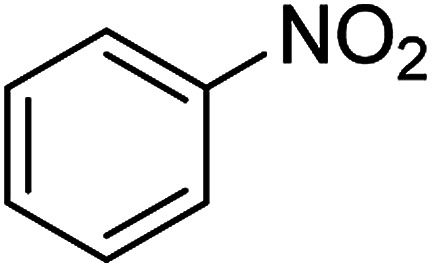	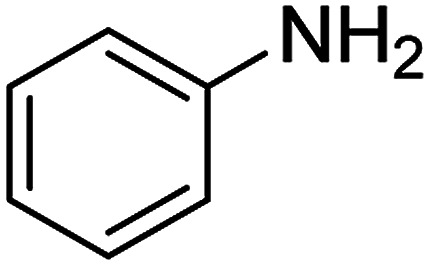	6	95	Liquid sample^62^	
2	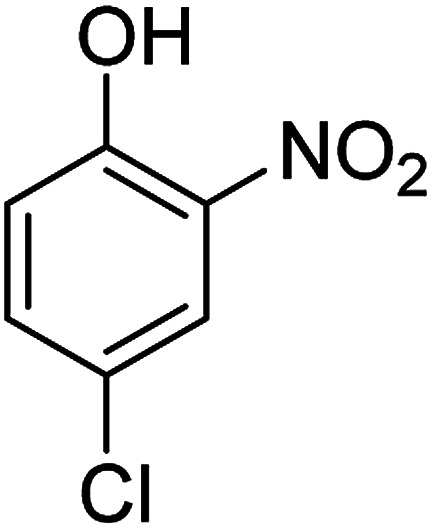	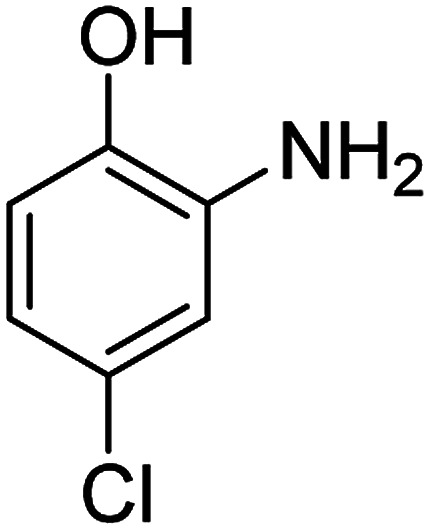	8	96	136–137	136–138 (ref. 63)
3	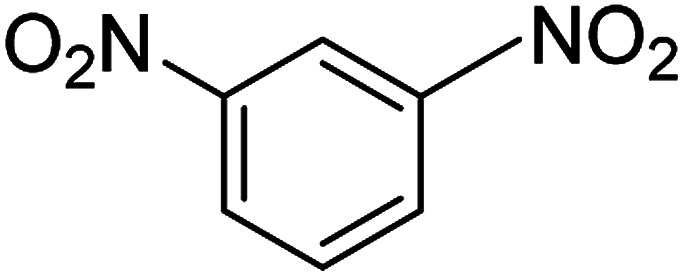	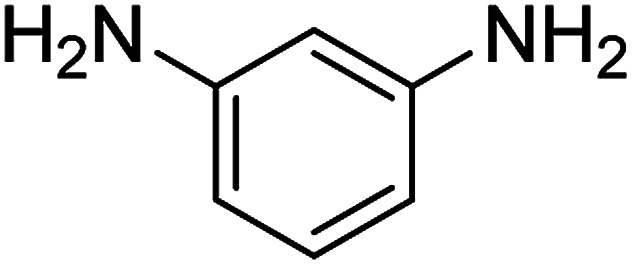	12	88	64–66	65–66 (ref. 64)
4	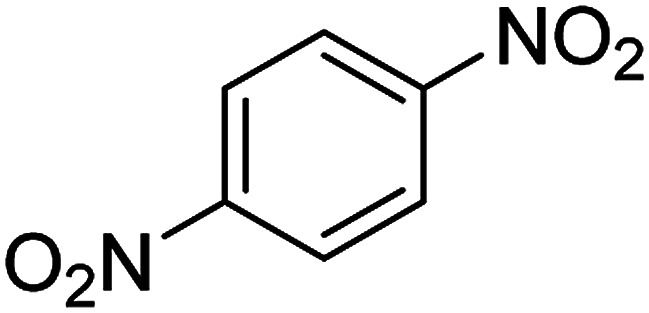	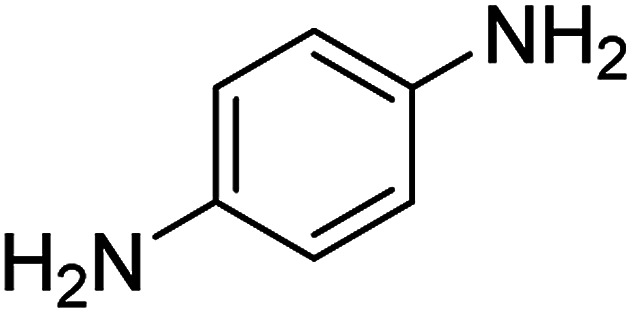	14	98	145–146	144–146 (ref. 65)
5	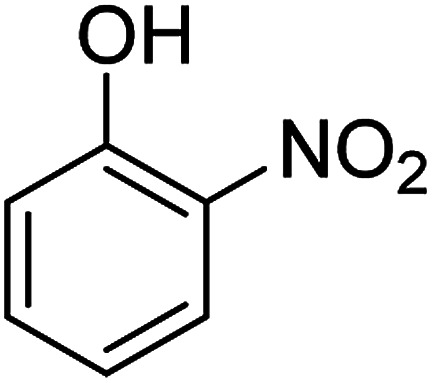	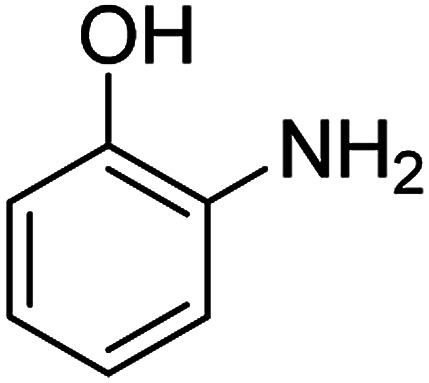	3	99	172–174	171–173 (ref. 63)
6	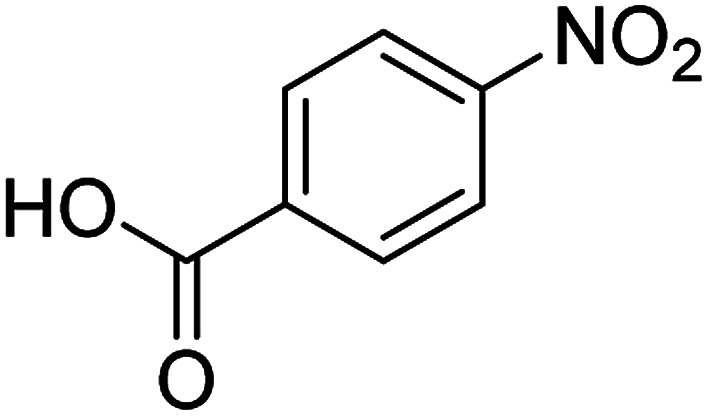	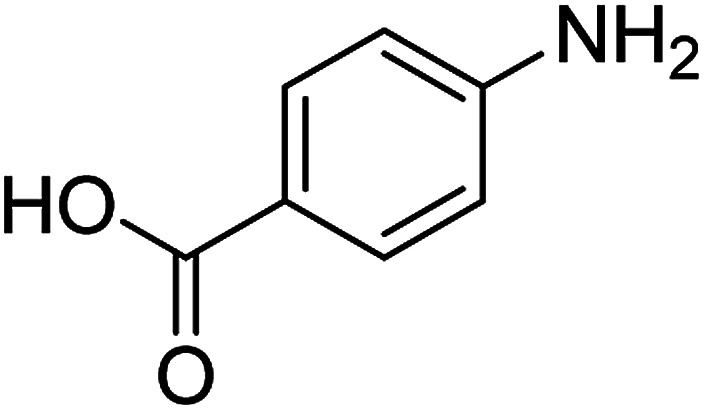	5	96	186–189	186–188 (ref. 62)
7	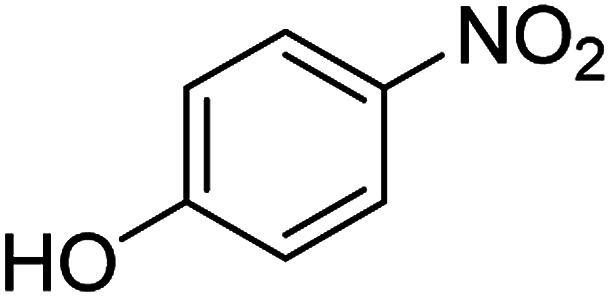	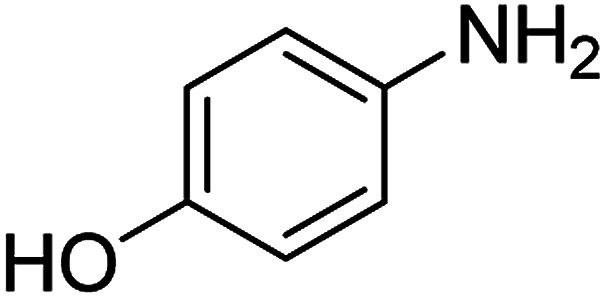	9	95	184–186	185–187 (ref. 66)
8	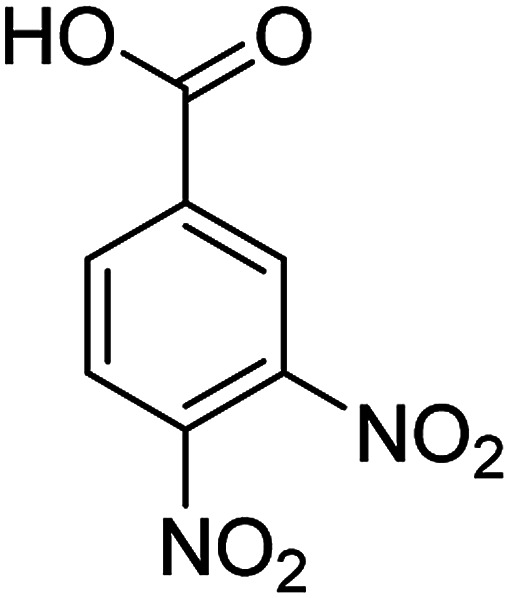	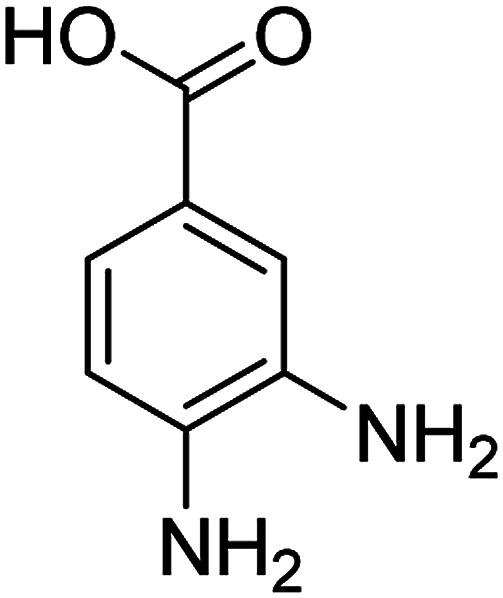	10	94	209–212	208–210 (ref. 67)
9	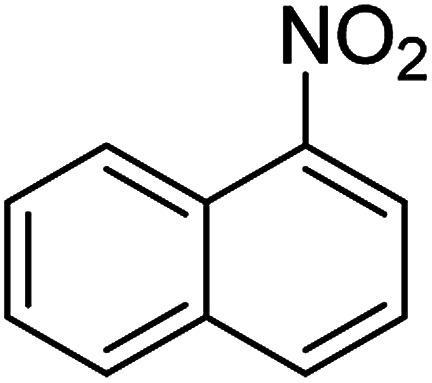	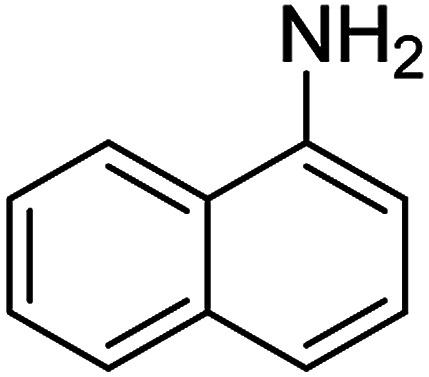	10	84	52–54	52–53 (ref. 68)
10	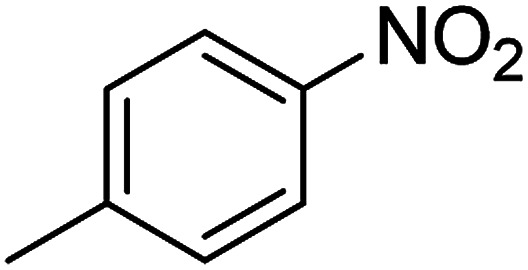	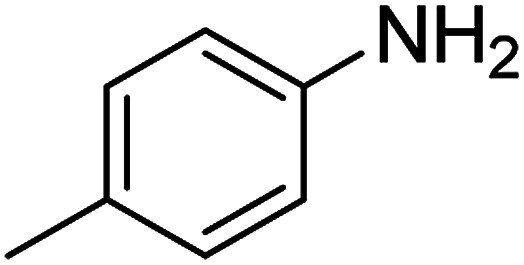	6	97	41–44	42–43 (ref. 69)

#### 2.3.3. Suggested mechanism

Based on the knowledge from the current report, Ag NPs are the leading catalytic site of the magnetic Ag@VP/CTS composite. They can facilitate the reduction reaction in a short reaction time. The catalytic process is described through the electron transfer system. The foremost advantage of the Ag NPs is their significant electronic interactions with the heteroatoms like oxygen. Hydrazine hydrate is an electron reducing agent with a significant supply of electron.^70^ Thus, the leading reason of the reduction of the NB derivatives is the dehydration process, which occurs because of the electron transfer between the hydrazine hydrate and the Ag NPs. After the electron transfer process occurred, the hydrogen atoms of the hydrazine hydrate spontaneously attack nearby NB molecules and produce the reduced non-hazardous form.^71^ In our previous report, a schematic of the plausible mechanism has been submitted that can properly justify the passing route by the Ag@VP/CTS catalytic system.^21^

#### 2.3.4. Recyclability effect of the nanocomposite catalytic system

**Fig. 5:**
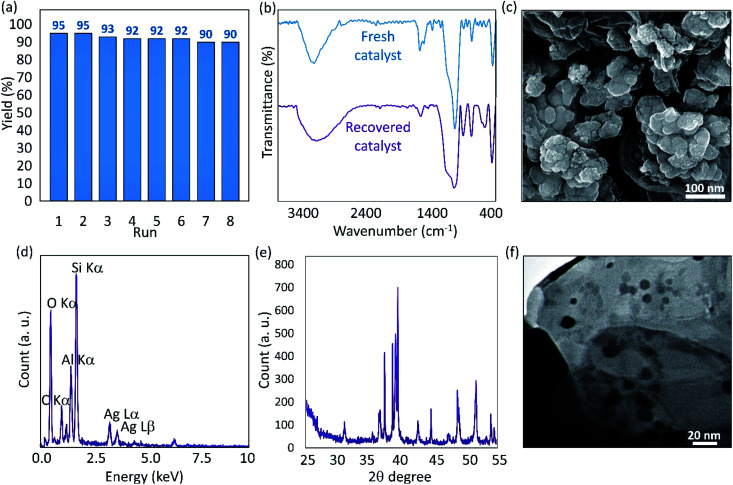
(a) Recyclability diagram, (b) the FT-IR spectra of fresh and recovered catalyst, (c) SEM image (d) EDX spectrum, (e) XRD pattern, and (f) TEM image of the recovered Ag@VP/CTS nanocatalyst.

In order to explore reusability of the fabricated Ag@VP/CTS catalytic system, the particles were magnetically collected from the reaction mixture, and prepared for further reactions. After separation of the particles, they were well washed with the distilled water for several times and dried in an oven. The fabricated Ag@VP/CTS was applied for the eight successive times in the model reaction, which is the reduction of nitrobenzene. As depicted in Fig. 5a, monitoring the catalytic process confirms that the reaction yield has not been significantly changed. Fig. 5b indicates the FT-IR spectrum of the recovered Ag@VP/CTS composite comparing with the spectrum of the fresh particles. As can be seen, emerging the sharp peak at ∼1100 cm^−1^ proves that the internal networks of the VP particles (silica and alumina) have not been damaged during the recycling process. Only, the intensity of the peak appeared at ∼1650 cm^−1^ (belonging to C=O) has been a bit reduced that may be due to separation of a part of CTS network. From the SEM imaging (Fig. 5c), it is figured out that particles agglomeration is occurred after an 8-time recycling. As discussed, since the VP particles are inherently magnetic, they are prone to attract each other and make bigger masses. This happening may be the main contributor to the reduction of the catalytic performance during the eight times recycling. The EDX analysis shown in panel (d) of the Fig. 5 proved that all of the essential elements were present after recycling. Also, the XRD pattern of the recovered Ag@VP/CTS catalytic system was prepared (Fig. 5e) to investigate any possible changes in the structural phases. As is observed, there was no noticeable change in comparison with the fresh catalyst (Fig. 4a), only the intensity of the appeared peaks has a bit changed. To investigate the particle size of the combined Ag NPs after the recycling process, the TEM image of the recovered Ag@VP/CTS was prepared. As Fig. 5f illustrates, the spherical morphology of the Ag particles with the mean size of *ca.* 17 nm has been maintained during the recycling processes. In comparison with the fresh particles (Fig. 3e), the number of the Ag NPs distributed on the VP/CTS substrate seems to be reduced. It may be another reason for the observed reduction in the catalytic performance after an 8-time recycling. To obtain more confirmation on this claim, the inductively coupled plasma optical emission spectroscopy (ICP-OES) was performed on a sample of the supernatant after eighth time of the particles usage, and it was revealed that 0.423 ppm of Ag element exist in the sample. It means that the leaching of the Ag element from the catalytic system is an inevitable event, but this partial leaching does not have any significant effects on the catalytic performance.

#### 2.3.5. Comparison of the prepared Ag@VP/CTS with other catalytic systems

In this section, we intend to make a quick comparison between our presented nanocatalyst and some of the previously reported systems that included Ag NPs and have been utilized for the conversion of the NBs to their aniline analogues. As Table 3 demonstrates, there are several advantages for the prepared Ag@VP/CTS composite in comparison with the other species: (1) generally, magnetic systems are highly preferred due to their convenient separation process. As discussed in the previous sections, VP component inherently includes the magnetic property due to the presence of the iron element in its structure. (2) Significant conversion (95%) of the NB derivatives has been observed during a short contact time (5 min), whereas in the most cases (Table 3, entries 1, 2, 3, and 5) the reaction time exceeded 2 hours. (3) In the design and preparation stage, this is important to use the low cost materials with high degrees of biocompatibility and biodegradability. In comparison with the system reported in Table 3 (entry 4), although the obtained results by the reported catalytic system are great, but exploitation of the abundant natural materials (like VP rock) has always been preferred due to its economic advantage. In addition of the mentioned excellences, the use of the light-weight substrates with the porous structure causes the catalyst particles to be well dispersed in the reaction medium, and as a result the total performance will be increased.

**Table 3:** Previously reported nanoscale catalytic systems based on the Ag element, applied for the conversion of the NB derivatives to their aniline analogues

Entry	Catalyst	Condition	Cat. amount^a^ (mg)	Time (min)	Conversion (%)	Ref.
1	Ag–Ni core/shell NPs	80 °C, KOH, IPA^b^	50	150	95	72
2	Ag–rGO^c^ composite	Visible light irradiation	50	240	98	73
3	Ag–MPTA-1^d^ composite	80 °C, NaOH, under N_2_	250	600	97	74
4	Ag–SBA-15^e^ composite	NaBH_4_, r.t.	10	6	95	75
5	Ag–mNC^f^ composite	NaBH_4_, r.t.	5	150	97	76
6	Ag–VP/CTS^g^ composite	N_2_H_4_·H_2_O, 70 °C	30	5	95	—

a Per 1.0 mmol of the NB compound.

b Isopropyl alcohol (IPA).

c Reduced graphene oxide.

d Poly-triallylamine (MPTA-1).

e SBA-15 is a mesoporous silica.

f Mesoporous melamine-N-doped carbon (mNC).

g This work.

## 3. Experimental section

### 3.1. Materials and physical characterizations

Altogether, the applied chemicals as well as the utilized apparatuses in the current report are listed in the ESI† part.

### 3.2. Practical approaches

#### 3.2.1. Synthesis of VP

First, 2.5 g of the obtained pumice powder was grinded by ball-milling technique for 1 h in 50 Hz. Afterwards, grinded pumice was added to the crucible and went through the calcination procedure for 5 h at 400 °C. After cooling down to room temperature, 1.5 g of the obtained pumice was moved to the round bottom flask (50 mL) and by utilizing an ultrasonic bath, it was dispersed in prepared HCl solution at room temperature (0.1 M, 20 mL). Under the same condition, the bottom flask was stirred for another 12 h. Employing an external magnet, VP was separated, and washed for a couple of times with distilled water. Finally, it was dried over oven heat (60 °C) for 24 h.

#### 3.2.2. Preparation of VP/CTS

VP/CTS was synthesized by 0.01 g of CTS powder and 30 mL of deionized water to the round bottom flask (100 mL) and stirred at room temperature till a clear bleached solution was gained. Afterward, 1.0 g of the prepared VP was dispersed in the flask *via* the ultrasonication process, and the reaction mixture was stirred continuously at room temperature for an additional 5 h. VP/CTS was magnetically collected, washed with distilled water, and dried (at 60 °C) for 24 h.

#### 3.2.3. Preparation of Ag@VP/CTS composite

Obtained VP/CTS (0.6 g) was dispersed in deionized water (20 mL) and transferred to a round bottom flask (50 mL). AgNO_3_ (1.0 g, 5.9 mmol) was dispersed in the flask *via* ultrasonic at room temperature. By maintaining the same condition, it was stirred for 5 h. The magnetic composite was separated by an external magnet, washed with distilled water, and dried (as mentioned in the previous section).

### 3.3. Spectral information aimed at the selected composites

4-Aminophenol (Table 2, entry 7): white solid, ^1^H NMR (500 MHz, DMSO): *δ* (ppm) = 4.38 (2H, s, NH_2_), 6.42–6.44 (2H, d, *J* = 10 Hz, H–Ar), 6.48–6.50 (2H, d, *J* = 10 Hz, H–Ar), 8.37 (1H, s, OH).

## 4. Conclusions

In this report, a heterogeneous organic–inorganic catalytic system was introduced and applied successfully in the conversion of hazardous NBs to nontoxic aniline derivatives. Overall, Ag NPs were successfully stabilized on the surface of biocompatible ingredients. This catalytic system contains natural components, VP with inherently significant magnetic property and CTS polymeric strands. Ag NPs are the main catalyst sites. They can facilitate the reduction reaction of NB derivatives with significant yield, in short reaction time under mild condition. The foremost advantage of magnetic catalysts is that they can be conveniently isolate from the reaction mixture. VP has great magnetic property along with structural stability, which results in significant reusability. This well-designed catalyst is based on green principles since all the ingredients are from natural components.

## Conflicts of interest

There are no conflicts of interest.

## Supplementary Material

RA-010-D0RA08376C-s001
