# Expression of Concern: Convenient conversion of hazardous nitrobenzene derivatives to aniline analogues by Ag nanoparticles, stabilized on a naturally magnetic pumice/chitosan substrate

**DOI:** 10.1039/d3ra90111d

**Published:** 2023-11-08

**Authors:** Reza Taheri-Ledari, Seyedeh Shadi Mirmohammadi, Kobra Valadi, Ali Maleki, Ahmed Esmail Shalan

**Affiliations:** a Catalysts and Organic Synthesis Research Laboratory, Department of Chemistry, Iran University of Science and Technology (IUST) Tehran 16846-13114 Iran maleki@iust.ac.ir +98-21-73021584 +98-21-77240640-50; b Central Metallurgical Research and Development Institute (CMRDI) P.O. Box 87 Helwan Cairo 11421 Egypt a.shalan133@gmail.com; c BCMaterials, Basque Center for Materials, Applications and Nanostructures, Martina Casiano, UPV/EHU Science Park Barrio Sarriena s/n Leioa 48940 Spain

## Abstract

Expression of Concern for ‘Convenient conversion of hazardous nitrobenzene derivatives to aniline analogues by Ag nanoparticles, stabilized on a naturally magnetic pumice/chitosan substrate’ by Reza Taheri-Ledari *et al.*, *RSC Adv.*, 2020, **10**, 43670–43681, DOI: https://doi.org/10.1039/D0RA08376C.

The Royal Society of Chemistry is publishing this expression of concern in order to alert readers that concerns have been raised regarding the reliability of the EDX data in Fig. 2b. An investigation is underway, and an Expression of Concern will continue to be associated with the article until a final outcome is reached.

Laura Fisher

2nd November 2023

Executive Editor, *RSC Advances*

## Supplementary Material

