# Supplementary material for: Convenient conversion of hazardous nitrobenzene derivatives to aniline analogues by Ag nanoparticles, stabilized on a naturally magnetic pumice/chitosan substrate
Source: RSC Adv. 2020 Dec 8;10(71):43670–81. doi: 10.1039/d0ra08376c (PMC9058380; doi:10.1039/d0ra08376c)
Supplement: RA-010-D0RA08376C-s001 [file RA-010-D0RA08376C-s001.pdf]

Electronic Supplementary Material (ESI) for RSC Advances.
This journal is © The Royal Society of Chemistry 2020

Supplementary Information

Convenient conversion of hazardous nitrobenzene derivatives to aniline analogues by Ag nanoparticles, stabilized on the naturally magnetic pumice/chitosan substrate

Reza Taheri-Ledari[1, +], Seyedeh Shadi Mirmohammadi[1, +], Kobra Valadi[1], Ali Maleki[1,*] and Ahmed Esmail Shalan[2,3,*]

[1] *Catalysts and Organic Synthesis Research Laboratory, Department of Chemistry, Iran University of Science and Technology (IUST), Tehran 16846-13114, Iran.*

[2] *Central Metallurgical Research and Development Institute (CMRDI), P.O. Box 87, Helwan, Cairo 11421, Egypt.*

[3] *BCMaterials, Basque Center for Materials, Applications and Nanostructures, Martina Casiano, UPV/EHU Science Park, Barrio Sarriena s/n, Leioa 48940, Spain.*

**Corresponding authors. (A. M) Tel.: +98 21 77240640-50; fax: +98 21 73021584. E-mail address: maleki@iust.ac.ir; (A. E. S.) E-mail: a.shalan133@gmail.com*

[+] *Authors contributed equally.*

Author's ORCIDs:

Reza Taheri-Ledari: https://orcid.org/0000-0002-6511-9411

Ali Maleki: https://orcid.org/0000-0001-5490-3350

Ahmed Esmail Shalan: https://orcid.org/0000-0002-3424-1609

Content	Page
**Figure S1.** H and C NMR spectra and spectral data of aniline	**S2**
**Figure S2.** H and C NMR spectra and spectral data of benzene-1,4-diamine	**S3**
**Figure S3.** H and C NMR spectra and spectral data of 2-amino-4-chloro-phenol	**S4**
**Figure S4.** H and C NMR spectra and spectral data of 2-aminophenol	**S5**
**Figure S5.** H and C NMR spectra and spectral data of 4-amino-benzonic acid	**S6**
**Figure S6.** H and C NMR spectra and spectral data of 4-amino-phenol	**S7**
**Figure S7.** H and C NMR spectra and spectral data of benzene-1,3-diamine	**S8**
**Figure S8.** H and C NMR spectra and spectral data of 2-Naphthylamine	**S9**
**Figure S9.** H and C NMR spectra and spectral data of p-toluidine	**S10**
**Figure S10.** H and C NMR spectra and spectral data of 4-chloroaniline	**S11**
Materials and equipment used in this study	**S12**

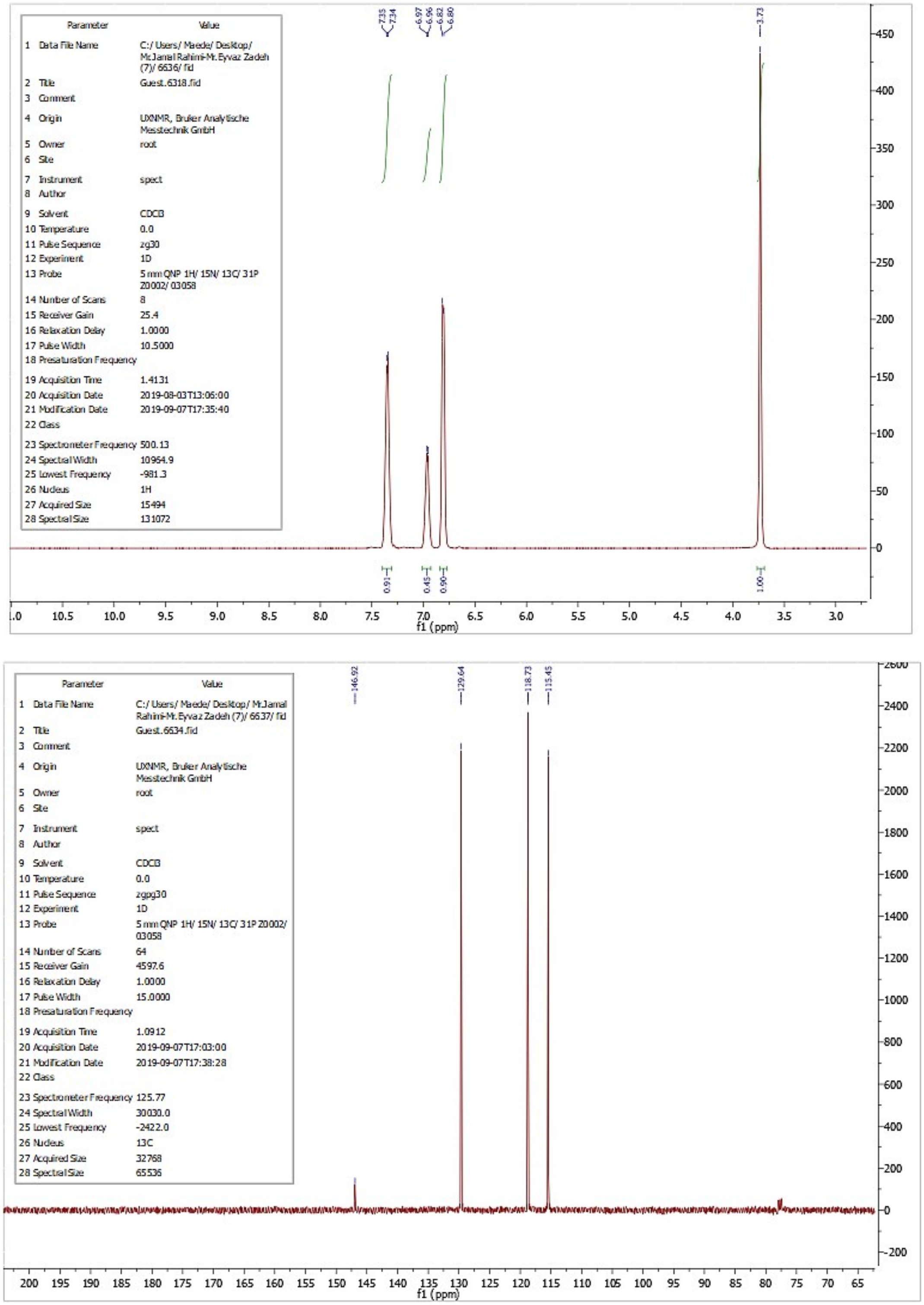

1. aniline (**Table 2 – Entry 1)**

^{1}H NMR (500 MHz, $CDCl_3$) δ 3.73 (s, 2 H), 6.80-6.82 (d, J = 10 Hz, 2 H), 6.93-6.99(m, 1H), 7.34-7.35 (d, J = 5 Hz, 2 H). ^{13}C NMR (500 MHz, $CDCl_3$) δ 146.92, 129.64, 118.73, 115.45.

Figure S1. H and C NMR spectra and spectral data of aniline.

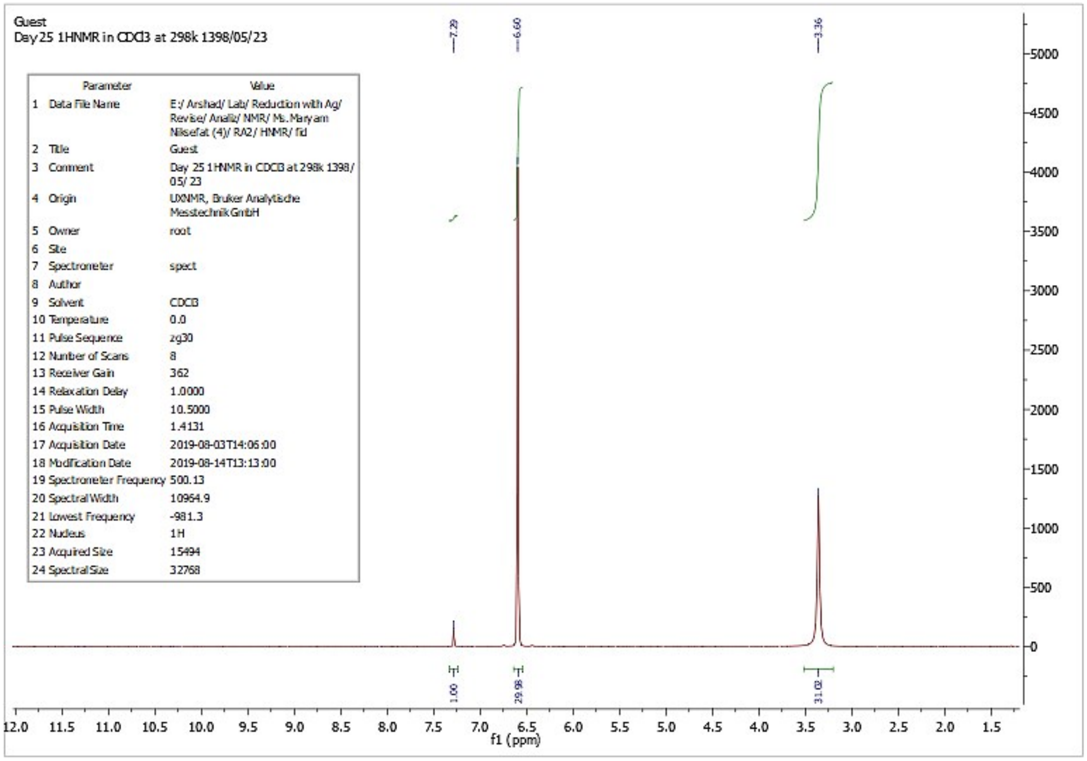

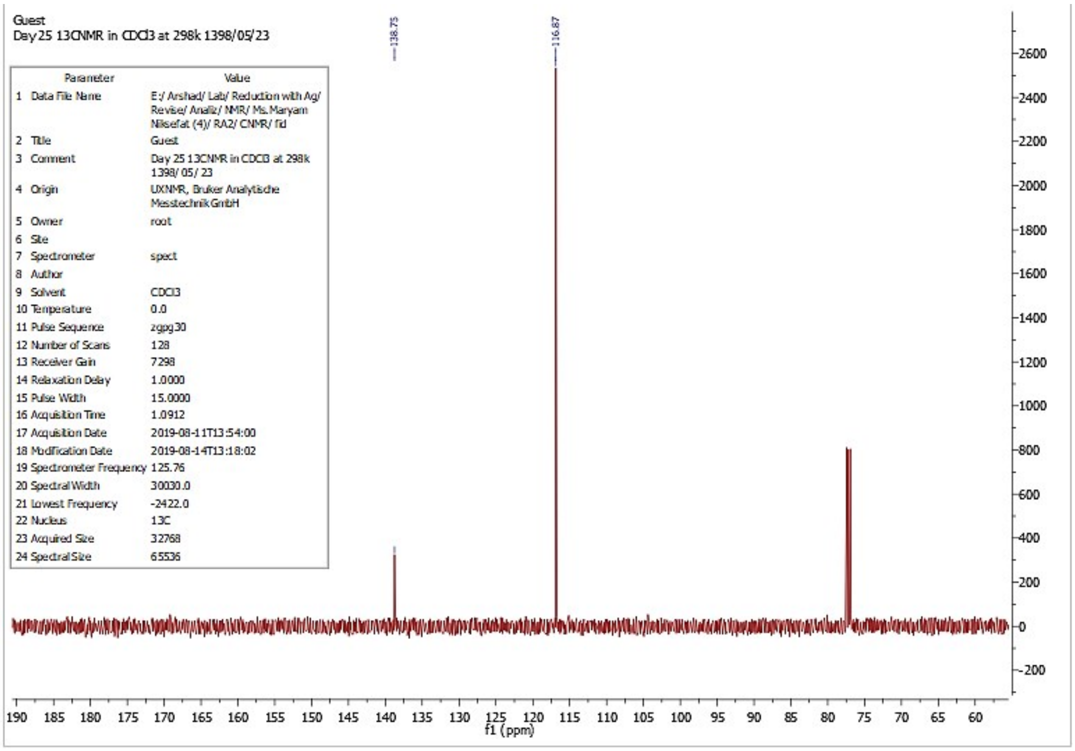

2. benzene-1,4-diamine (**Table2 – Entry 4)**

^{1}H NMR (500 MHz, $CDCl_3$) δ 6.59 (s, 4 H), 3.36 (s, 4 H). ^{13}C NMR (500 MHz, $CDCl_3$) δ 138.75, 116.87.

Figure S2. H and C NMR spectra and spectral data of benzene-1,4-diamine.

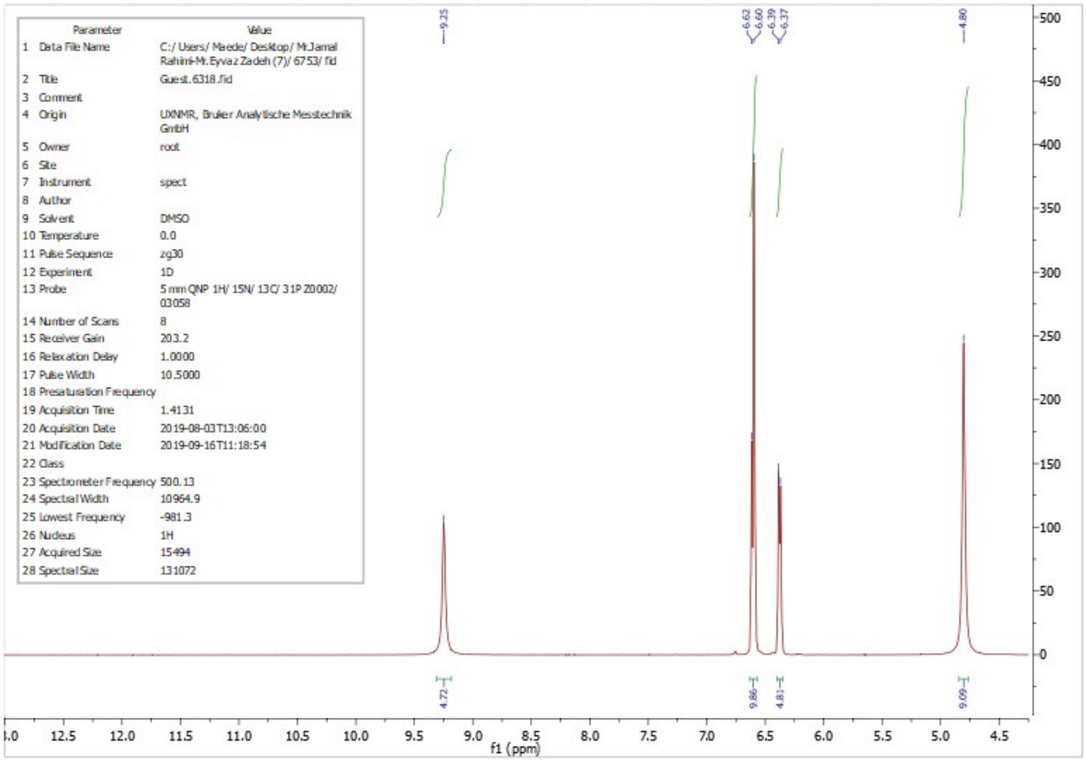

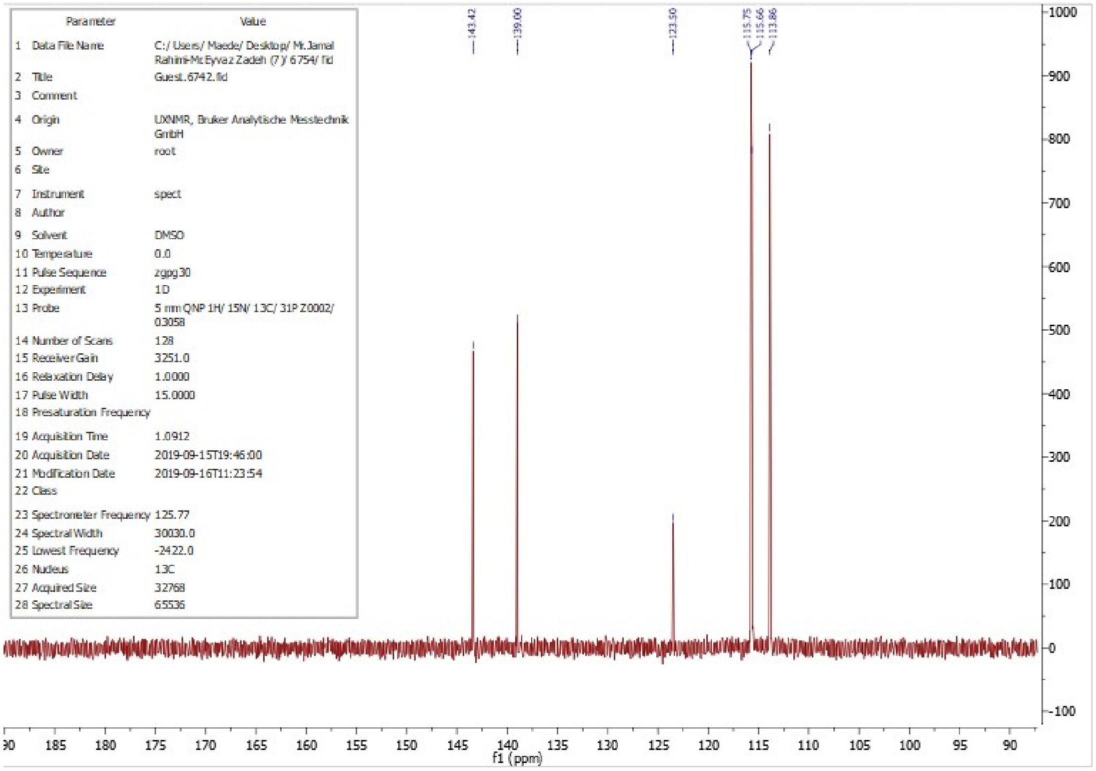

3. 2-amino-4-chloro-phenol (**Table 2 – Entry 2)**

^{1}H NMR (500 MHz, DMSO) δ 9.25 (s, 1H), 6.61 (m, 1H), 6.61 (m, 1H), 6.38 (m, 1H), 4.80 (s, 2H). ^{13}C NMR (500 MHz, DMSO) δ 143.42, 139.00, 123.50, 115.75, 115.66, 113.86.

Figure S3. H and C NMR spectra and spectral data of 2-amino-4-chloro-phenol.

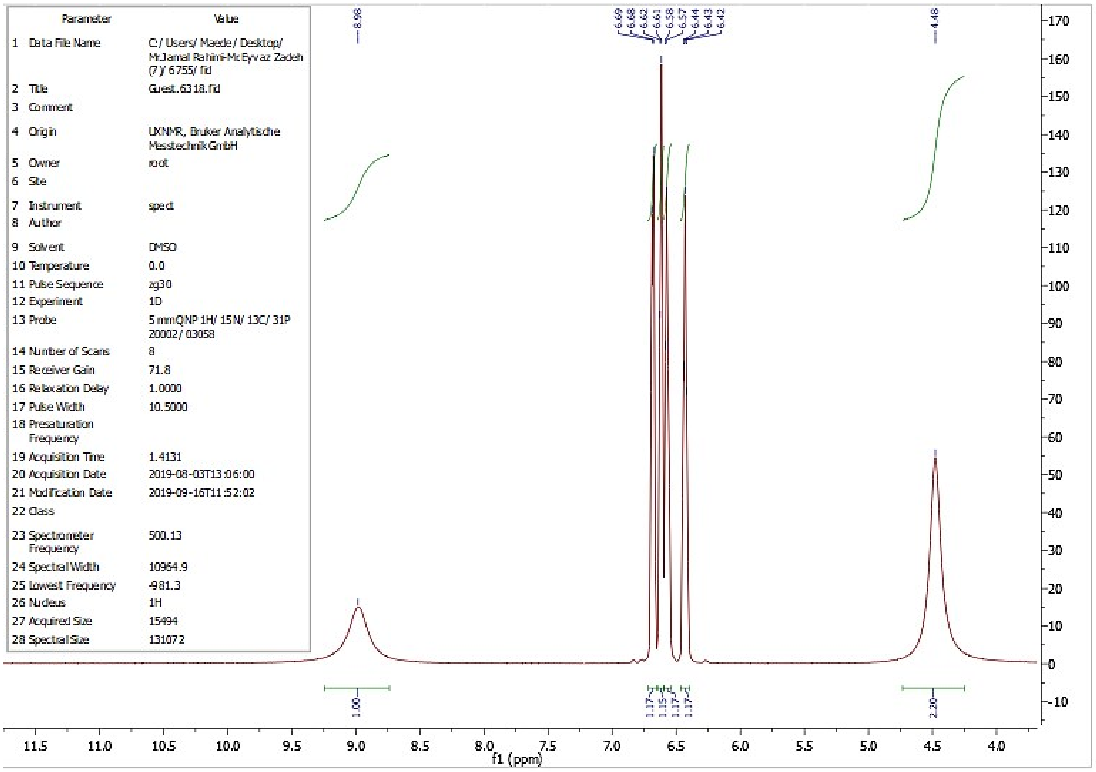

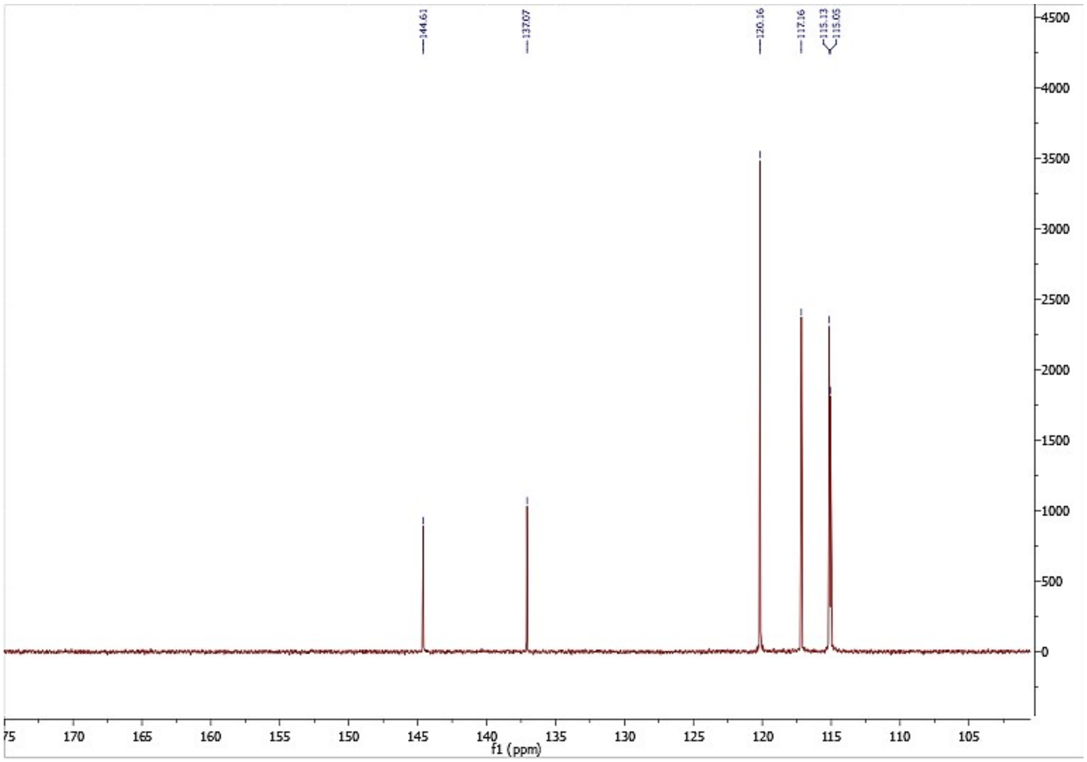

4. 2-aminophenol (**Table 2 – Entry 5)**

1H NMR (500 MHz, DMSO) δ 8.98 (s, 1H), 6.68 (m, 1H), 6.61 (m, 1H), 6.57 (m, 1H), 6.43 (m, 1H), 4.48 (S, 2H). ^{13}C NMR (500 MHz, DMSO) 144.51, 137.07, 120.16, 117.16, 115.13, 115.05.

Figure S4. H and C NMR spectra and spectral data of 2-aminophenol.

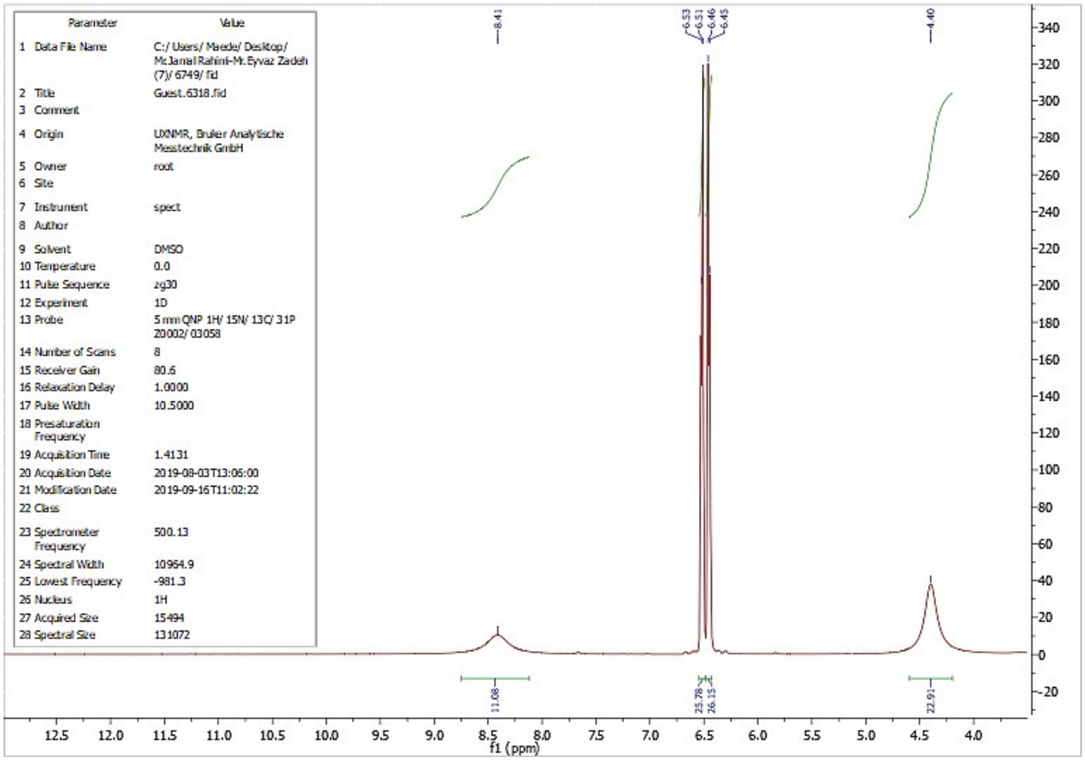

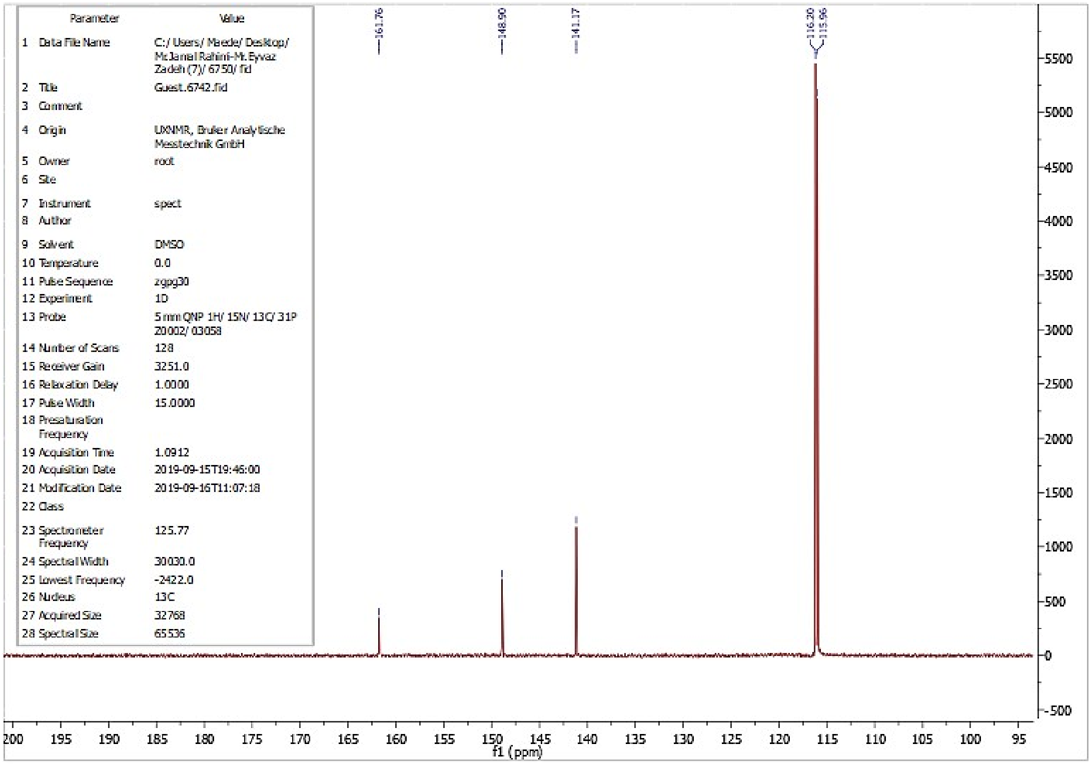

5. 4-amino-benzonic acid (**Table 2 – Entry 6)**

^{1}H NMR (500 MHz, DMSO) δ 8.41 (s, 1H), 6.53-6.51 (d, J = 10 Hz, 2H), 6.46-6.45 (d, J = 5 Hz, 2H), 4.40 (s, 2H). ^{13}C NMR (500 MHz, DMSO) δ 161.76, 148.90, 141.17, 116.20, 115.96.

Figure S5. H and C NMR spectra and spectral data of 4-amino-benzonic acid.

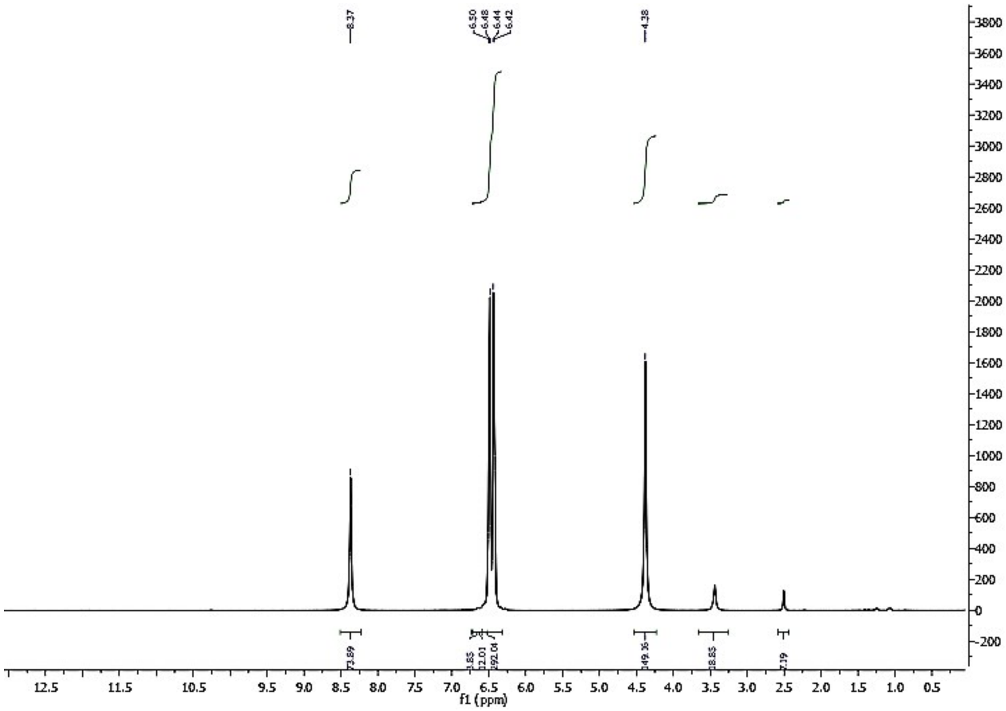

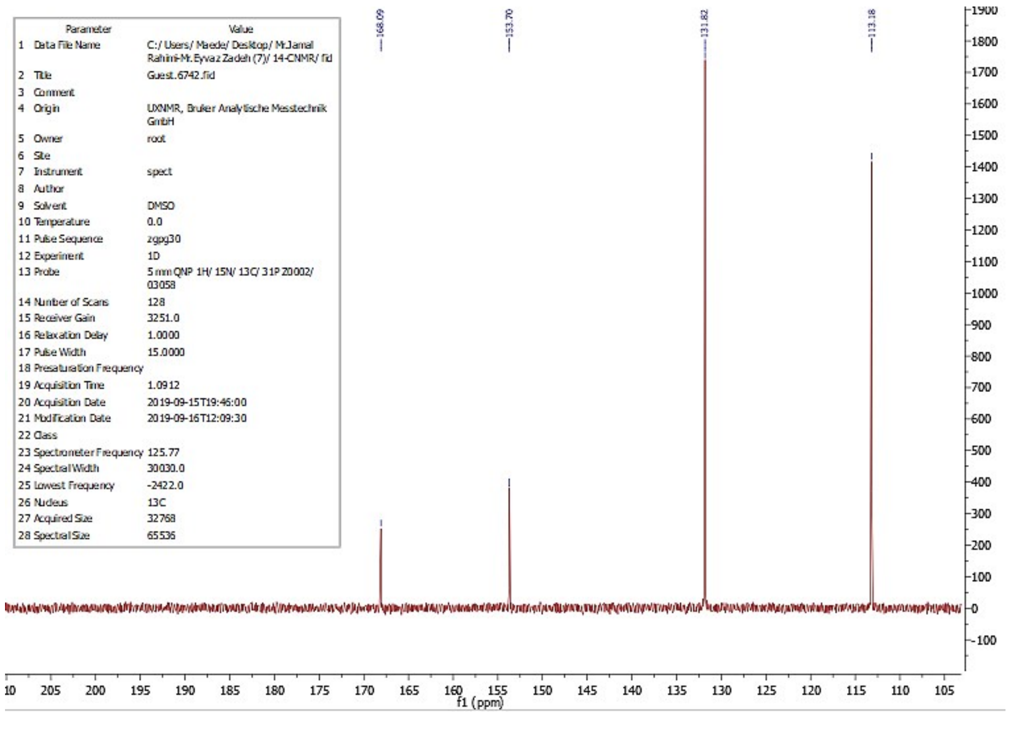

6. 4-amino-phenol (**Table 2 – Entry 7)**

4-Aminophenol (Table 3, Entry 6): white solid, ^{1}H NMR (500 MHz, DMSO): δ (ppm) = 4.38 (2H, s, NH_2), 6.42–6.44 (2H, d, J=10 Hz, H–Ar), 6.48–6.50 (2H, d, J=10 Hz, H–Ar), 8.37 (1H, s, OH). ^{13}C NMR (500 MHz, DMSO) δ 168.09, 153.70, 131.82, 113.18.

Figure S6. H and C NMR spectra and spectral data of 4-amino-phenol.

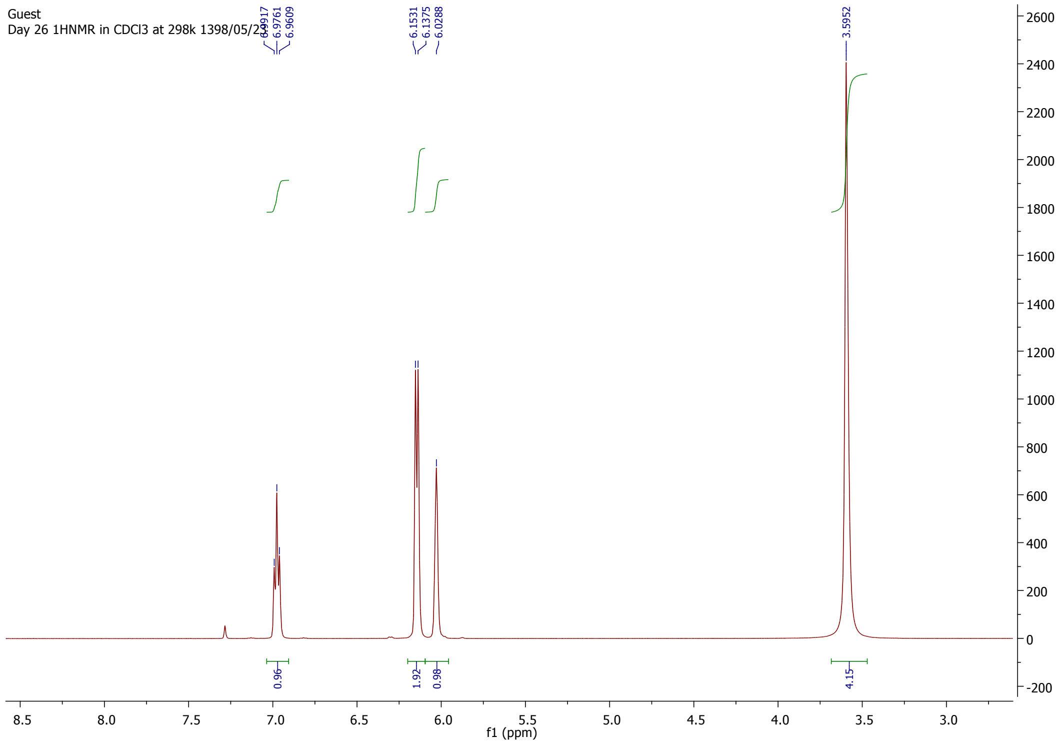

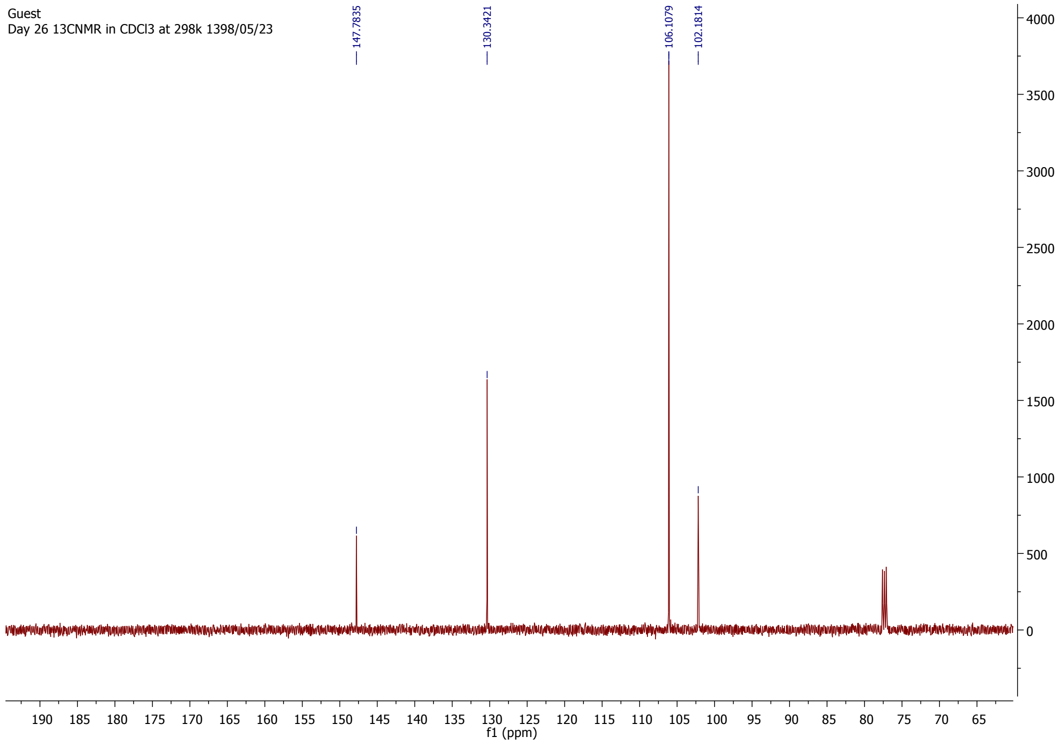

7 - benzene-1,3-diamine (**Table 2 – Entry 3)**

^{1}H NMR (500 MHz, $CDCl_3$) δ 6.96-6.99 (m, 1 H), 6.15–6.13 (d, J = 7.8 Hz, 2 H), 6.03 (s, 1 H), 3.59 (s, 4 H). ^{13}C NMR (500 MHz, $CDCl_3$) δ 147.78, 130.34, 106.25, 102.18.

Figure S7. H and C NMR spectra and spectral data of benzene-1,3-diamine.

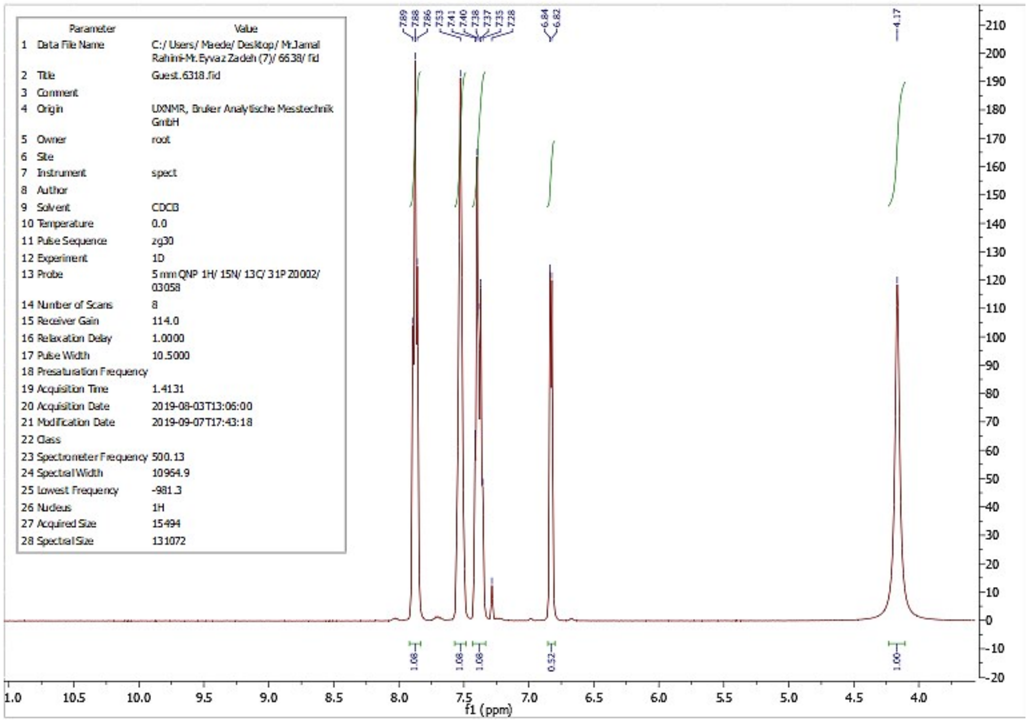

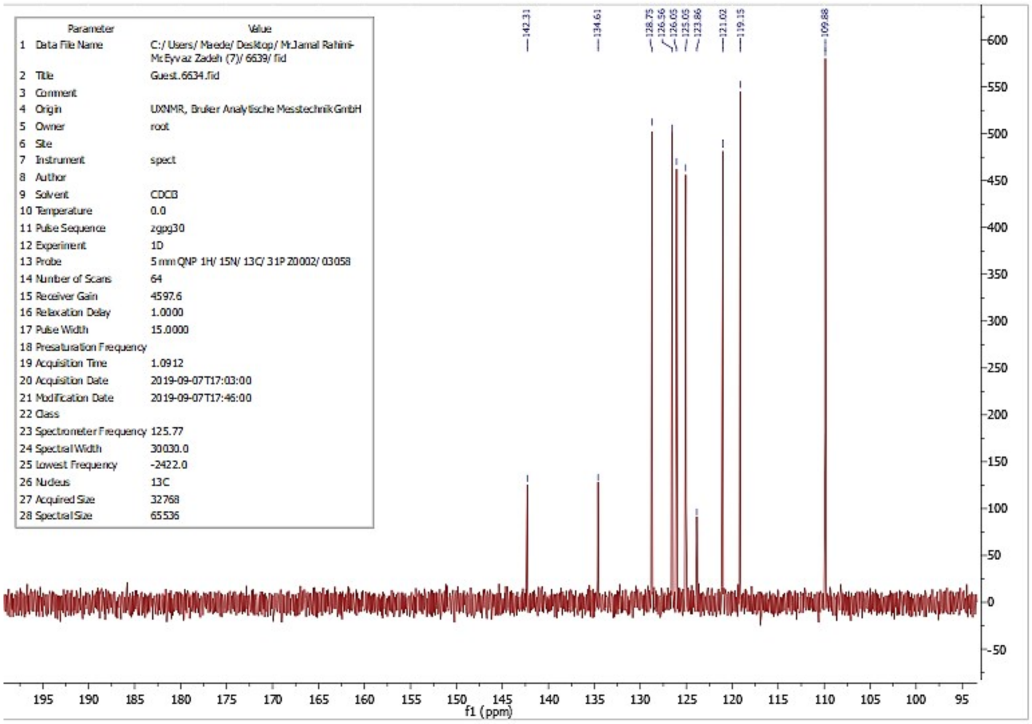

8. 2-Naphthylamine (**Table 2 – Entry 9)**

^{1}H-NMR (500 MHz, $CDCl_3$): δ = 7.88 (m, 2H), 7.53 (m, 2H), 7.38 (m, 2H), 6.83 (m, 1H), 4.17 (s, 2H). ^{13}C-NMR (500 MHz, $CDCl_3$): δ = 142.31, 134.61, 128.75, 126.56, 126.05, 125.05, 123.86, 121.02, 119.15, 109.88.

Figure S8. H and C NMR spectra and spectral data of 2-Naphthylamine.

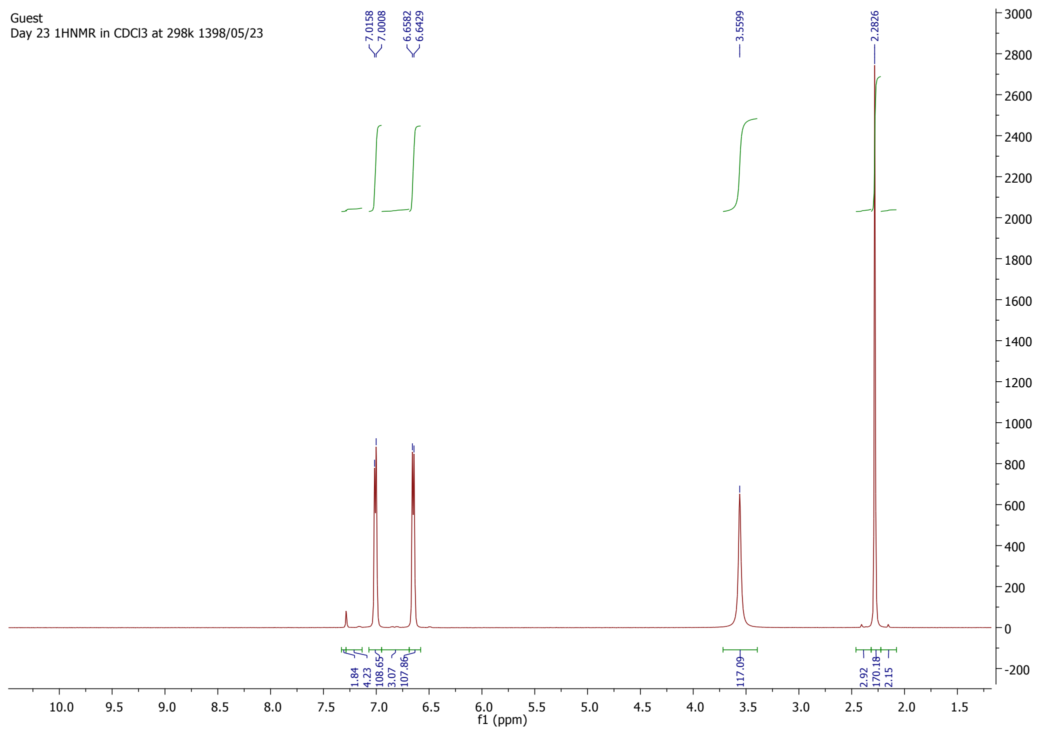

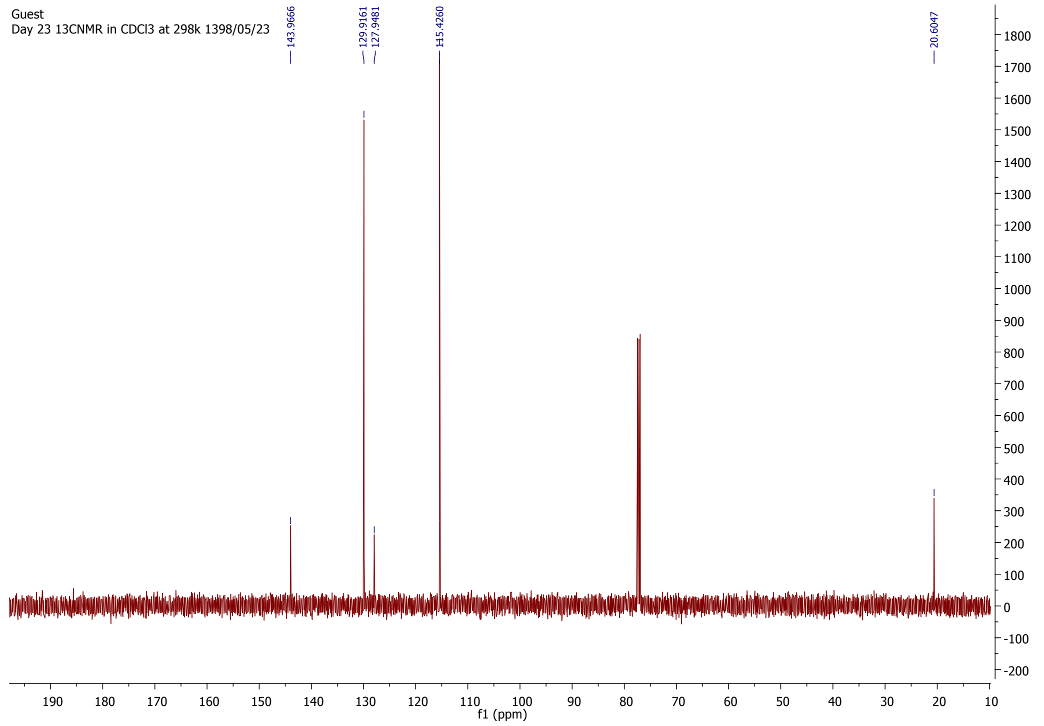

9- p-toluidine (**Table 2 – Entry 10)**

^{1}H NMR (500 MHz, $CDCl_3$) δ 2.28 (s, 3H), 3.56 (s, 2H), 6.64-6.66 (d, J = 10 Hz, 2H), 7.00-7.02 (d, J = 10 Hz, 2H). ^{13}C NMR (500 MHz, $CDCl_3$) δ 20.60, 115.43, 127.96, 129.92, 143.97.

Figure S9. H and C NMR spectra and spectral data of p-toluidine.

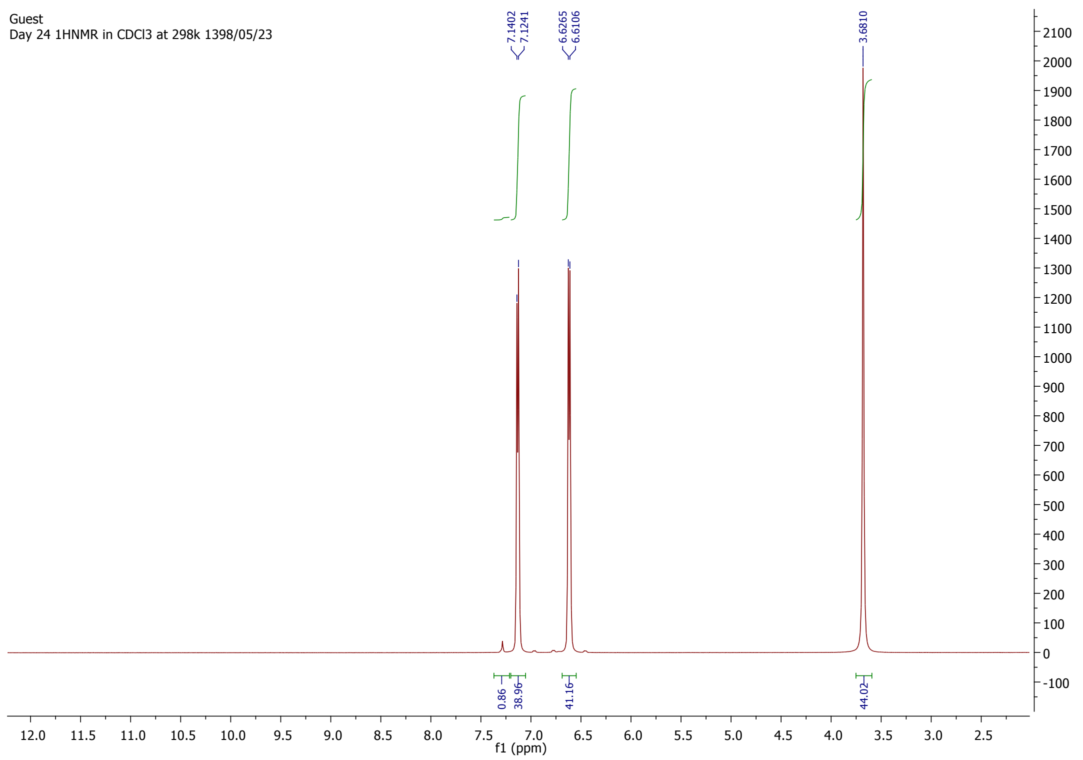

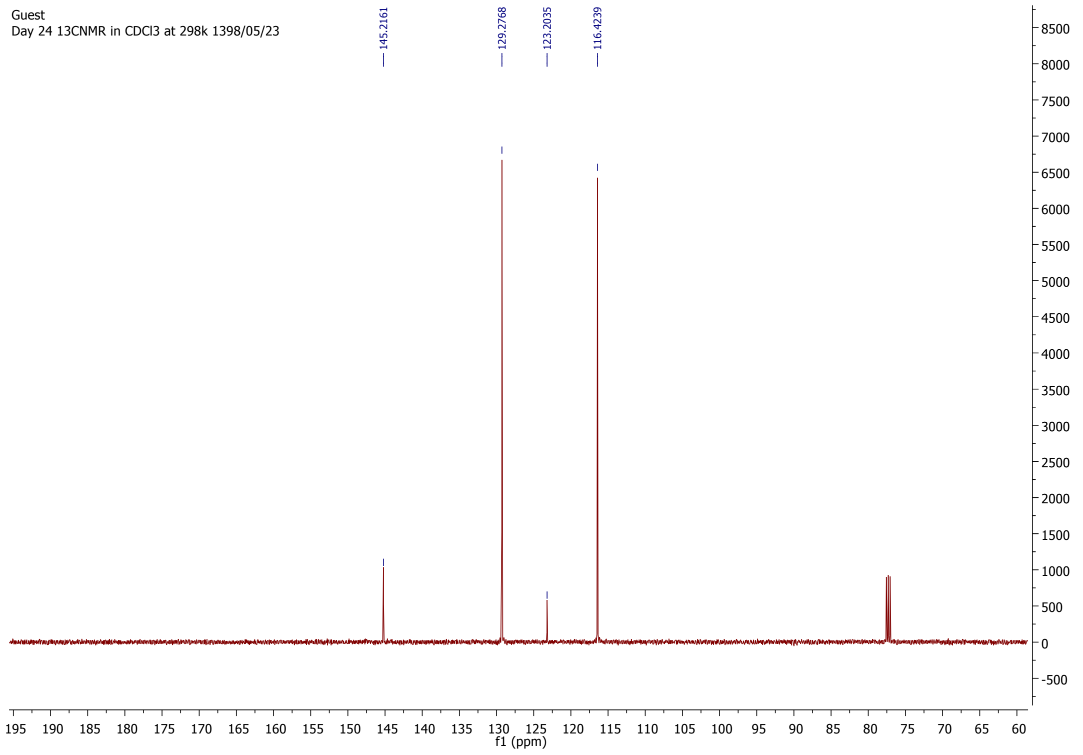

10- 4-chloroaniline (**Table 2– Entry 8)**

^{1}H NMR (500 MHz, $CDCl_3$) δ 3.68 (s, 2H), 6.61-6.63 (d, J = 10 Hz, 2H), 7.12-7.14 (d, J = 10 Hz, 2H).
^{13}C NMR (500 MHz, $CDCl_3$) δ 116.42, 123.20, 129.28, 145.22.

Figure S10. H and C NMR spectra and spectral data of 4-chloroaniline.

Materials and equipment:

All solvents, chemicals, and reagents were purchased from Merck and Sigma-Aldrich. Melting points were measured on an Electrothermal 9100 apparatus. The FT-IR spectra were taken with a Shimadzu IR-470 spectrometer, EDX spectra were recorded on Numerix DXP–X10P, the SEM images were prepared through Mira3 Tescan microscope, the elemental states were analyzed by X-ray photoelectron spectroscopy (XPS, ESCALAB Xiþ, and Thermo Scientific), XRD measurements were carried out by using a X' Pert Pro X-ray diffractometer operating at 40 mA, 40 kV, thermal analysis (TGA) was done by using of Bahr-STA 504 instrument under argon atmosphere, the magnetic properties of sample were detected at room temperature using a VSM (Meghnatis Kavir Kashan Co., Kashan, Iran), ^{1}H-NMR spectra were prepared with Varian Unity Inova 500 MHz. The statistical data of particle sizes from SEM imaging and XRD analysis were obtained by Digimizer and Highscore plus software, respectively. For ICP-OES investigations were performed on an Agilent, made in Germany.
